# Training Infrastructure as a Service

**DOI:** 10.1093/gigascience/giad048

**Published:** 2023-07-03

**Authors:** Helena Rasche, Cameron Hyde, John Davis, Simon Gladman, Nate Coraor, Anthony Bretaudeau, Gianmauro Cuccuru, Wendi Bacon, Beatriz Serrano-Solano, Jennifer Hillman-Jackson, Saskia Hiltemann, Miaomiao Zhou, Björn Grüning, Andrew Stubbs

**Affiliations:** Department of Pathology and Clinical Bioinformatics, Erasmus Medical Center, Dr. Molewaterplein 40, 3015 GD, Rotterdam, the Netherlands; School of Life Sciences and Technology, Avans University of Applied Sciences, Lovensdijkstraat 63, 4818 AJ Breda, the Netherlands; Queensland Cyber Infrastructure Foundation Ltd., The University of Queensland, St. Lucia, QLD 4072, Australia; University of the Sunshine Coast, Maroochydore, QLD 4558, Australia; Department of Biology, Johns Hopkins University, Baltimore, MD 21218, USA; Melbourne Bioinformatics, The University of Melbourne, Melbourne, VIC 3051, Australia; School of Life, Health & Chemical Sciences, The Open University, Milton Keynes MK7 6AA, UK; IGEPP, INRAE, Institut Agro, University of Rennes, 35000 Rennes, France; GenOuest Core Facility, University of Rennes, Inria, CNRS, IRISA, 35000 Rennes, France; Bioinformatics Grou, Department of Computer Science, University of Freiburg, 79110 Freiburg im Breisgau, Germany; School of Life, Health & Chemical Sciences, The Open University, Milton Keynes MK7 6AA, UK; Euro-Bioimaging ERIC Bio-Hub, EMBL, 69117 Heidelberg, Germany; Department of Biochemistry and Molecular Biology, Eberly College of Science, The Pennsylvania State University, State College, PA 16802, USA; Euro-Bioimaging ERIC Bio-Hub, EMBL, 69117 Heidelberg, Germany; Department of Pathology and Clinical Bioinformatics, Erasmus Medical Center, Dr. Molewaterplein 40, 3015 GD, Rotterdam, the Netherlands; School of Life Sciences and Technology, Avans University of Applied Sciences, Lovensdijkstraat 63, 4818 AJ Breda, the Netherlands; Bioinformatics Grou, Department of Computer Science, University of Freiburg, 79110 Freiburg im Breisgau, Germany; Department of Pathology and Clinical Bioinformatics, Erasmus Medical Center, Dr. Molewaterplein 40, 3015 GD, Rotterdam, the Netherlands

**Keywords:** Galaxy, training, teaching, remote training

## Abstract

**Background:**

Hands-on training, whether in bioinformatics or other domains, often requires significant technical resources and knowledge to set up and run. Instructors must have access to powerful compute infrastructure that can support resource-intensive jobs running efficiently. Often this is achieved using a private server where there is no contention for the queue. However, this places a significant prerequisite knowledge or labor barrier for instructors, who must spend time coordinating deployment and management of compute resources. Furthermore, with the increase of virtual and hybrid teaching, where learners are located in separate physical locations, it is difficult to track student progress as efficiently as during in-person courses.

**Findings:**

Originally developed by Galaxy Europe and the Gallantries project, together with the Galaxy community, we have created Training Infrastructure-as-a-Service (TIaaS), aimed at providing user-friendly training infrastructure to the global training community. TIaaS provides dedicated training resources for Galaxy-based courses and events. Event organizers register their course, after which trainees are transparently placed in a private queue on the compute infrastructure, which ensures jobs complete quickly, even when the main queue is experiencing high wait times. A built-in dashboard allows instructors to monitor student progress.

**Conclusions:**

TIaaS provides a significant improvement for instructors and learners, as well as infrastructure administrators. The instructor dashboard makes remote events not only possible but also easy. Students experience continuity of learning, as all training happens on Galaxy, which they can continue to use after the event. In the past 60 months, 504 training events with over 24,000 learners have used this infrastructure for Galaxy training.

Key points

- The private queue offered by most Training Infrastructure-as-a-Service (TIaaS) deployments ensures that courses run smoothly and efficiently.
- Infrastructure is generally complicated and difficult to set up, as well as at cross-purposes to instructors’ main focus.
- TIaaS provides “1-click” infrastructure for instructors that simplifies hosting courses.
- The dashboard enables remote training, allowing instructors to follow student progress.

## Findings

Training Infrastructure as a Service (TIaaS) has been in development since 21 June 2018 and 3 days later became a production service at Galaxy Europe on 24 June. Here we present the development and rationale for implementing this service.

### Background

With the large volume of bioinformatics data being generated, the availability of training for bioinformaticians and data scientists is not keeping up, resulting in a training gap [1].

The Galaxy platform [2] provides infrastructure suitable not only for data analysis but also for conducting trainings, as it provides a user-friendly web-based interface to command-line analysis tools. Teaching with Galaxy significantly decreases infrastructure preparation time for instructors [3]. With a wide range of tools (8,000+) across a broad range of scientific domains and preexisting popularity within the life sciences community, Galaxy is an ideal platform for training [3, 4].

In an attempt to address the training gap, the Galaxy community has, over the past several years, developed a large number of hands-on tutorials (300+)—covering bioinformatics and beyond—and made these materials Findable, Accessible, Interoperable, and Reusable (FAIR) [5, 6] and publicly available on the Galaxy Training Network (GTN) repository [7]. In order to run these tutorials at scale, one often needs access to significant resources. For example, the GTN’s most popular tutorial, “Reference-Based RNA-Seq Data Analysis,” uses the STAR aligner [8]. While such an ultra-fast aligner is ideal during training, as it reflects real-world analysis, it also consumes ≈32 GB of RAM at minimum. (It uses 90 GB of RAM in the default configuration on UseGalaxy.eu. Many tools have similar requirements; on UseGalaxy.eu, 83 tools require >64 GB RAM, and 151 require >32 GB, a limiting factor especially for smaller training infrastructures. Even with a large computer cluster, even moderate class sizes of 20 to 40 can still consume all of the available overhead.) Individual STAR jobs might execute successfully and quickly, but the infrastructure remains a limiting factor for events with a large number of participants, especially if the class is to remain on schedule. When jobs must queue due to throughput limitations, this negatively impacts a training’s timeline, to the detriment of learners.

While the instructor could potentially deploy their own private infrastructure, this requires additional knowledge, time, energy, and funds, all of which are significant barriers for bioinformatics instructors preparing to teach a course. There are numerous attempts to decrease the effort required to deploy a Galaxy server such as Laniakea [9], CloudLaunch [10], and AnVIL [11], but most of these require access to a public or private cloud and a compute budget. Given the presence of numerous large Galaxy deployments that offer compute and data storage for free, a solution that can leverage these existing centers of Galaxy and system administration experience is highly desirable.

Lastly, with the recent increase of remote and hybrid training—in which an instructor is streamed live to multiple locations—due to the COVID-19 pandemic, tracking student progress in a remote learning setting has became a significant issue. During one of the initial Gallantries project’s [12] hybrid training events, with 3 classrooms spread across Europe, we discovered that staying updated on student progress was one of the most significant pain points. Normally, instructors of hands-on lessons tend to wander around the classroom to check that students are not encountering difficulties or use the Carpentries-style [13, 14] method of red and green sticky notes to let students communicate whether things are going well or poorly. In hybrid events, this progress tracking is more difficult as on-site staff need to survey the room and report back centrally to the instructor, and this is near impossible in fully remote training events such as have been more prevalent during the past 3 years of the pandemic [15].

### Results

We initially developed TIaaS for the European Galaxy server [16], to solve the challenge of ensuring we could quickly set up a private queue for a single course or workshop. We achieved this by segregating student jobs onto a separate and dedicated group of compute nodes, based on their membership in a specific group in Galaxy.

We subsequently made TIaaS available for any training organizers to request free of charge. By reusing an existing public Galaxy server such as Galaxy Europe, which is backed by significant compute resources, the barriers for course organizers around infrastructure setup and maintenance costs of hosting a training event are removed. This centralization also reduced the infrastructure requirements, as training events are not highly concurrent and can share the same hardware when not running simultaneously.

**Figure 1:**
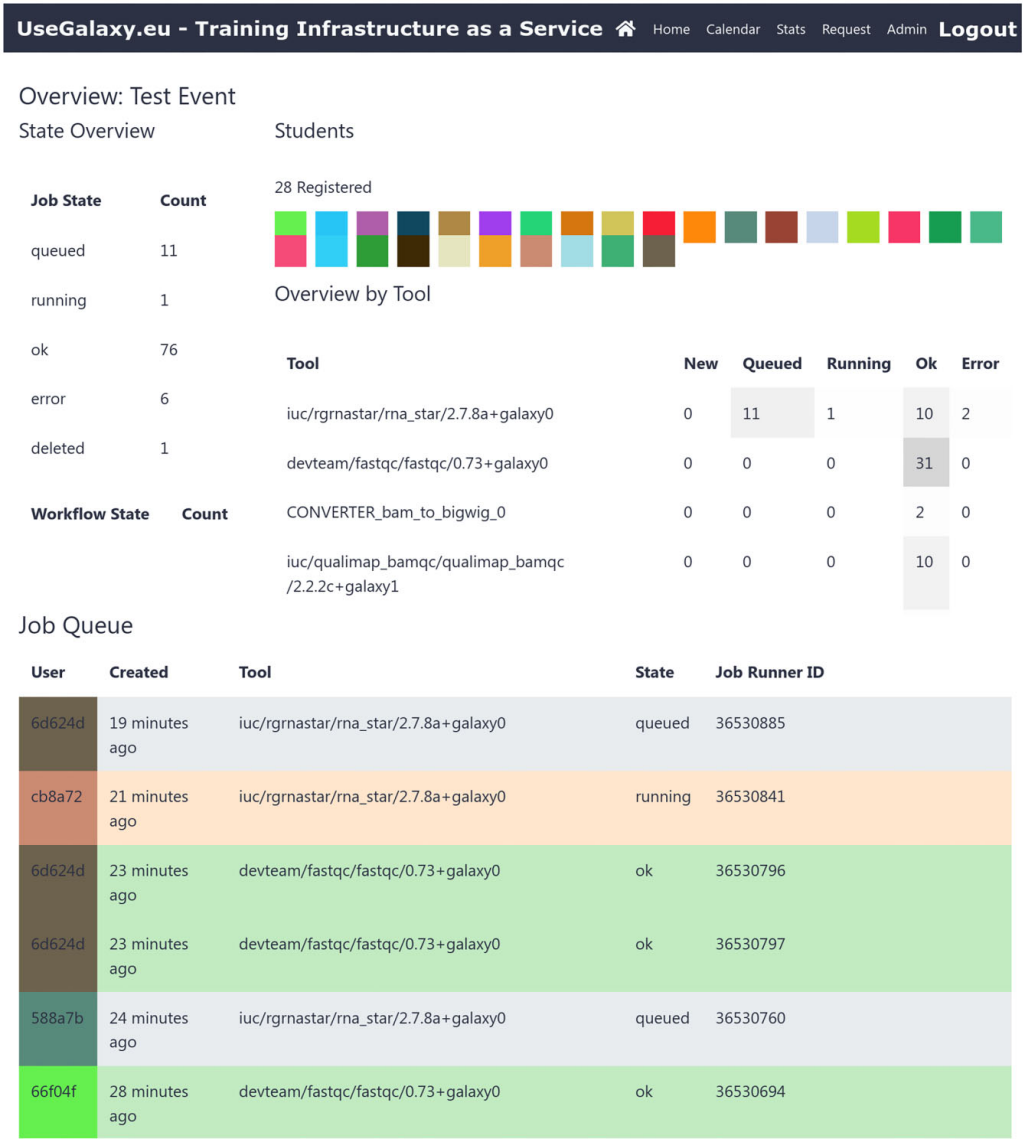
The top portion of the training dashboard page shows the status of the jobs in the past hours. A grayscale heatmap of the tools that were run indicates if everything is running smoothly or if there is anything the instructors should look into. As learners follow along and run different tools, these show up immediately in the dashboard, allowing instructors to identify if everyone has started or finished a specific step. The bottom portion shows the rest of the training dashboard, which lists jobs and workflows that were run, chronologically, color-coded first by user and second by the job status. Randomized colors and identifiers are used to protect user privacy.

When using TIaaS for a training event, a live dashboard (Fig. 1) becomes available to instructors, showing the status of participants’ jobs, providing visibility into student progress, and enabling instructors to flag potential issues that may benefit from additional discussion with learners. We have shared this service with the Galaxy training community to overwhelmingly positive feedback, anecdotally [17].

#### Deployment

The TIaaS system can be deployed on any Galaxy server and by its design is extremely flexible, allowing Galaxy administrators to customize the settings to fit their needs and compute infrastructure. TIaaS is currently deployed on all 3 major public Galaxy servers (Galaxy EU, Galaxy Australia, and Galaxy US) and numerous other smaller servers in public and private deployments. As compute infrastructures can be highly heterogeneous, we do not prescribe a single preferred method in which to preference training jobs. As a result, administrators have generally allocated private resources so jobs can run without delay, with the exception of one site that preferences jobs by scheduling rules.

TIaaS provides a good separation of responsibilities between instructors who are teaching and the server administrators responsible for Galaxy and the compute infrastructure, rather than requiring either group to be cross-trained.

#### Development

To create TIaaS (RRID: SCR_023200), we implemented 2 components: a web service and a default set of Galaxy job scheduling rules, which function together to present a private queue for users in specific Galaxy user groups. The web service enables registering requests for resources and an approval workflow for administrators. Additionally, it handles creating groups in Galaxy and adding members to those groups as needed.

The registration form provided by the web service allows instructors to submit requests for TIaaS resources. Anyone wishing to host a training or workshop occurring on the Galaxy platform is welcome to do so as there is no formal qualification process for Galaxy instructors. Within the TIaaS request form, they are asked to provide information about the training materials they will use and the expected number of participants. TIaaS coordinators or system administrators review these requests, using information about the class size, the tools used in the training materials, and the resource allocations of those tools on the infrastructure to estimate the required compute resources.

A typical request timeline looks like an instructor submitting a request with 1 or more weeks’ advance notice, as the TIaaS service will automatically reject requests that are made within a configurable length of time before the start of the course. This feature was added as a result of too many last-minute requests placing undue burden on administrators. In exceptional circumstances, administrators can manually add a training at a specific date. Most approved TIaaS requests are accepted (*n* = 371/397), with most requests happening 7 to 14 days ahead of the event (*n* = 75), while many occur in the last week (*n* = 62) or even 3 (*n* = 65), 4 (*n* = 46), or 5 (*n* = 39) weeks in advance.

If resources are available and any other site-specific criteria are met (e.g., any legal restrictions on what sort of trainings can be provided on grant-funded infrastructure), then the training can be approved. Next, administrators (optionally) deploy additional private compute resources or reallocate existing resources to course usage. Administrators can then provide instructors with a URL such as /join-training/test [18], which the instructor can share with learners.

Training participants access this URL at the start of the event, after which they are automatically registered in the TIaaS system without further user interaction and without instructors needing to manually manage group membership. This aids in user privacy as the instructor does not need to collect user e-mails to manage their group, and learners can opt in to joining the training group.

**Figure 2:**
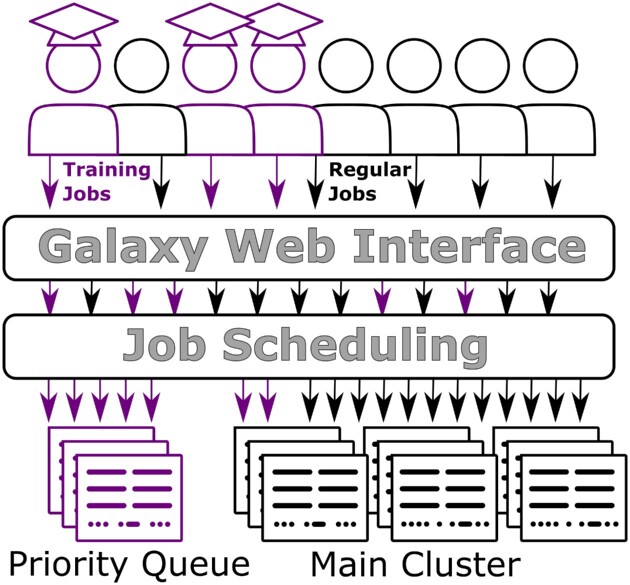
Schematic of the idealized TIaaS queuing system. Jobs are processed by the same Galaxy server, but when those jobs come from users in the training group, they receive special handling. These jobs are allowed to run on the private training resources (purple). If the training resource is full, these jobs can spill over to the main queue if necessary.

The job scheduler, once aware of the training group, will place any job run by someone in that training onto the private training nodes (Fig. 2).

During the course, instructors have access to the course dashboard, visualizing the progress of participants (Fig. 1), significantly improving the ability of instructors to monitor progress of the learners, especially in situations involving remote participants. The dashboard provides instantaneous, aggregated, and pseudonymized feedback for the instructors into how the learners are progressing. It has also simplified progress tracking in hybrid trainings, which were previously very labor intensive due to the necessity of maintaining insight into potential issues across multiple locations. This required per-site helpers to regularly update the instructor as to how participants were progressing. With the training dashboard, however, the instructor is no longer dependent on these communications from the satellite locations but can monitor progress via the dashboard themselves, in real time. Instructors can see which analysis steps are completed and by how many of the participants. Whenever there are any issues (e.g., failed jobs), they can use this information to decide whether they need to pause or reexplain the step in more detail.

The most similar system the authors could find, which could be used for the same goal of monitoring student progress, is currently implemented in Nextflow. “Nextflow Tower” [19], which permits launching and subsequently monitoring pipelines, could be used to cover a similar case of making sure students meet certain progress markers. However, given that it works at the workflow level and not the individual step level, it may be less suited to the sort of ad hoc analysis skills that are commonly taught using Galaxy and more suited to either advanced students or those trainings that involve running predefined workflows. Snakemake has a similar, albeit single-user, project called Panoptes that provides similar workflow tracking [20], with the same downsides as Nextflow Tower, relative to TIaaS.

#### Usage

**Figure 3:**
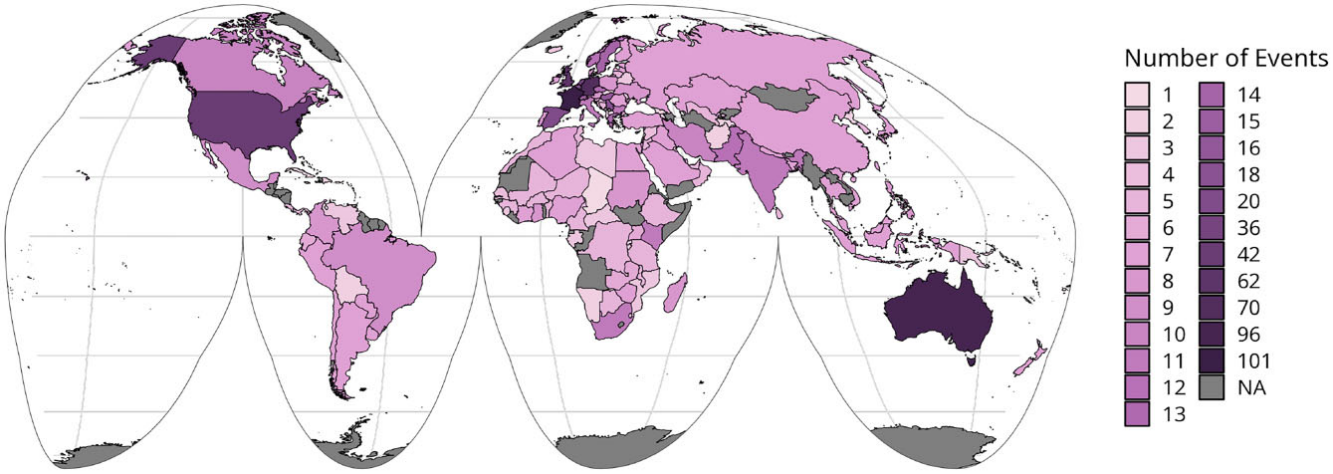
Map of countries targeted by TIaaS events. This combines 2 datasets: the statistics provided by the Application Programming Interfaces (APIs) of the 4 discussed TIaaS servers and a set of corrections from course registration data for the Smörgåsbord event series. This correction is needed as the authors did not sufficiently fill out the TIaaS form when they requested resources for the Smörgåsbord event, choosing to specify only a single country, which would otherwise result in potential undercounting of countries actually targeted by TIaaS managed events.

**Figure 4:**
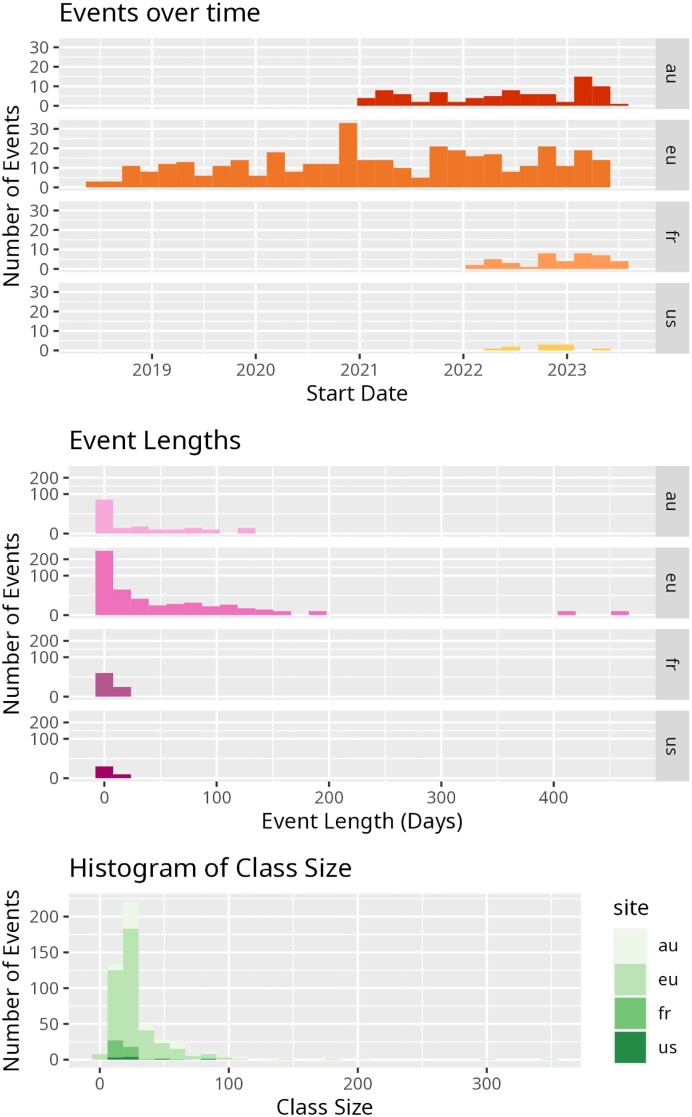
Since its introduction, it has grown into a well-used service over the past 4 years. There have been 438 training events, primarily hosted by the Australian and European servers, which are both very involved in training. Event length distribution in days is extremely heavily skewed to very short events, with a long tail of semester-long courses using the platform. Event sizes show a similar distribution; most classes are small, while 7 extremely large courses (>500 participants) were filtered from this graph as outliers. These courses are more like Massive Open Online Courses (MOOCs) than traditional in person courses.

Since the introduction of TIaaS in 2018, it has seen nearly constant use with 504 trainings occurring on the platform, all across the world (Figs. 3 and 4). Everything from 1-day workshops for bioinformaticians to multimonth courses for high school and university students have all been hosted by these 4 TIaaS deployments, covering topics as wide-ranging as SARS-CoV-2 analysis, imaging, proteomics, and machine learning. All of this infrastructure has been provided for free across these 4 instances in the European Union, France, the Americas, and Australia, thanks to the various grants supporting their associated Galaxy deployments.

Class sizes have ranged considerably from the median of 25 participants (interquartile range = 19) to a maximum of 1,500 registrants for a fully asynchronous (self-paced) course. Most courses were short training events with a median of 2 days, but some ran for multiple months like a number of high school or university courses that used TIaaS over the entire semester. The variability in administrator deployments of TIaaS can allow it to accommodate a wide range of teaching scenarios; for some courses, large resources may be allocated like the Galaxy Community Conferences, where the big 3 Galaxies configured TIaaS with considerable resources to permit local and remote synchronous training, all the way to semester-long courses, which may not necessitate a large allocation.

TIaaS has been successfully scaled to extremely large and highly geographically distributed events. The GTÑ project successfully used it for a Spanish-language bioinformatics course spanning the Americas and Europe [21], while the 2 Smörgåsbord events used TIaaS for a weeklong, global, asynchronous course with trainees across 111 countries [22].

In a hackathon environment, TIaaS has allowed large dataset (single-cell RNA sequencing) manipulation within group projects in remote courses with up to 30 participants performing unique analyses [23]. It has successfully supported an introduction to a bioinformatics course at a remote-learning, entrance exam–free alternative education institution (The Open University), as well as industry courses, allowing them to test out Galaxy as a collaborative working environment before making decisions on consortium platform use.

## Methods

### Implementation

TIaaS was written in Python with the Django framework [24]. It has been designed from the start to have a very limited scope: provide a form to register events, an approval flow for administrators, management of user groups and roles in the Galaxy database, and the status dashboard. Service metrics are exposed via a Prometheus [25] endpoint at /tiaas/metrics [26], for visualization and alerting.

Instructors can visit (/tiaas/new) to register a new event and request resources. When submitted, this form is stored in the associated database, and administrators can view the requested training events and approve or reject them using the built-in Django admin interface. When users visit their training URL (/join-training/<id>), the system accesses their Galaxy session cookie, which is present as TIaaS is deployed at a path below Galaxy, and decodes it turning it into a Galaxy identity. This identity is then automatically associated with a Galaxy group named after the training (e.g., training-<id>), which is created on demand.

When visiting the dashboard (Fig. 1), the training ID is extracted from the URL (e.g., “test” from https://usegalaxy.eu/join-training/test/status), and all jobs, in the past 0 to 6 hours, from those users are presented in a pseudonymized manner.

#### Overview pages

Information over the status of the TIaaS system is provided via the interface. The calendar page made with Vue.js [27] (e.g., https://usegalaxy.eu/tiaas/calendar/) shows upcoming training events, as well as their details, if one is logged in as a TIaaS administrator. This is complemented by the stats view (e.g., https://usegalaxy.eu/tiaas/stats/), which shows overall system statistics, giving funding agencies and staff a live view of the impact their service is having on the global community.

#### Scheduling

**Figure 5:**
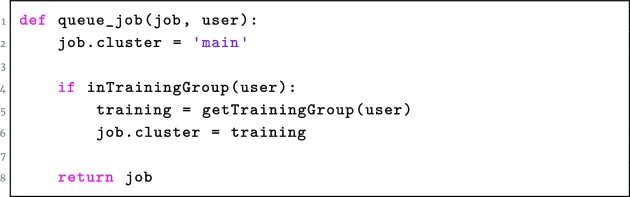
Pseudocode representing how TIaaS jobs are typically processed and allocated to a private queue.

**Figure 6:**
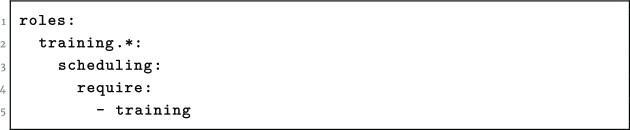
YAML-formatted TPV configuration that schedules jobs coming from users with a training role to any machines labeled as training nodes.

When a job is submitted by a user in a training group, the Galaxy instance’s job scheduling system reads the user’s groups and roles, and if any of these include something prefixed with training-, then this is converted to a job scheduler–specific requirement string (Figs. 5 and 6). Ideally, these are scheduled to prefer training nodes and spill over to the main queue if training nodes are full, but this feature is dependent on specific scheduler capabilities.

In HTCondor, this can be accomplished by preventing regular jobs from running on training nodes (e.g., a node’s configuration including Requirements=(GalaxyGroup == training-nld) || (GalaxyGroup == training-aus)) and then allowing training jobs to run on training nodes and preferring those nodes via configuration (e.g., a submit description including +Group=”training-aus, training-nld”).

Slurm, in contrast, requires either using Total Perspective Vortex’s (TPV’s) notion of machine tags to separate jobs into those specific groups of machines or simply manually selecting a reservation in which to run the training jobs, with –partition=training-nld.

Alternatively, these can be rewritten for the modern TPV [28] scheduler that is now being used at all 3 large UseGalaxy servers as seen in (Fig 6.)

### Flexible deployments

As the Galaxy community has largely settled on Ansible for deployment of Galaxy and related components, an Ansible role was produced for deploying the TIaaS service. A few known deployments make their configuration public, and as such, we can see what choices each administrator made. One of the motivating factors in TIaaS’s design was such flexibility, and this advantage is seen directly in those deployments.

*Galaxy Europe* uses it with HTCondor and job rules that allow spillover to the main cluster; new machines are brought up in an OpenStack cluster specifically for training events and destroyed afterward. Each machine is tagged with an HTCondor attribute indicating which training it belongs to, and the job rules (visible in https://github.com/usegalaxy-eu/infrastructure-playbook/pull/447/files) use that to enable access to those machines and a preference for them.

*Galaxy Australia* has a separate “training cluster” in their OpenStack deployment and routes all training jobs to the single shared cluster (visible in https://github.com/usegalaxy-au/infrastructure/tree/57cd80030d72929c263955e895079d6ac25aa24f/files/galaxy/dynamic_job_rules/production/total_perspective_vortex; note the training role and destinations tagged training).

*Galaxy US* takes a different approach, lacking additional clusters but having an efficient queuing system that can properly pack jobs based on walltimes; they instead artificially limit the runtime, memory, and CPU resources allocated to users running jobs within a TIaaS group.

*Avans Hogeschool* uses TIaaS in an internal Galaxy where they provide no preferential treatment and just use the dashboard to follow students’ progress (visible at https://github.com/Avans-ATGM/infrastructure/commit/11fa1ed38a7ed6640eafeca2ace8bb73e189301e).

## Availability of Source Code and Requirements

- Project name: Training Infrastructure as a Service
- SCR_023200
- bio.tools ID: tiaas
- Project homepage: https://github.com/galaxyproject/tiaas2/
- Admin training manual:  https://gxy.io/GTN:T00022
- Teacher training manual:  https://gxy.io/GTN:T00286
- Programming language(s): Python, Vue.js
- Operating system(s): Unix
- License:  GNU AGPL-3.0

## Supplementary Material

giad048_GIGA-D-23-00035_Original_Submission

giad048_GIGA-D-23-00035_Revision_1

giad048_GIGA-D-23-00035_Revision_2

giad048_Response_to_Reviewer_Comments_Original_Submission

giad048_Response_to_Reviewer_Comments_Revision_1

giad048_Reviewer_1_Report_Original_Submission Azza Ahmed -- 3/26/2023 Reviewed

giad048_Reviewer_2_Report_Original_Submission Elizabeth Ryder -- 4/3/2023 Reviewed

## Data Availability

All code is open source and available on GitHub [29, 30]. Snapshots of our code and other data further supporting this work are openly available in the *GigaScience* repository, GigaDB [31].

## Abbreviations

GTN: Galaxy Training Network; TIaaS: Training Infrastructure as a Service; FAIR: Findable, Accessible, Interoperable, Reusable; TPV: Total Perspective Vortex; API: Application Programming Interface; MOOC: Massive Open Online Course.
