# Supplementary material for: Training Infrastructure as a Service
Source: Gigascience. 2023 Jul 3;12:giad048. doi: 10.1093/gigascience/giad048 (PMC10316688; doi:10.1093/gigascience/giad048)
Supplement: giad048_GIGA-D-23-00035_Revision_2 [file giad048_giga-d-23-00035_revision_2.pdf]

GigaScience

Training Infrastructure as a Service

--Manuscript Draft--

**Manuscript Number:**	GIGA-D-23-00035R2
**Full Title:**	Training Infrastructure as a Service
**Article Type:**	Technical Note
**Funding Information:**	Bundesministerium für Bildung und Forschung (031 A538A/A538C RBC) — Not applicable<br>Deutsche Forschungsgemeinschaft (SFB 992/1 2012) — Not applicable<br>Bundesministerium für Bildung und Forschung (031L0101B/031L0101C de.NBI-epi) — Dr Björn Grüning<br>National Human Genome Research Institute (2U24HG006620) — Not applicable<br>Erasmus+ (2020-1-NL01-KA203-064717) — Dr Andrew Stubbs
**Abstract:**	Background: Hands-on training, whether in bioinformatics or other domains, often requires significant technical resources and knowledge to set up and run. Instructors must have access to powerful compute infrastructure that can support resource-intensive jobs running efficiently. Often this is achieved using a private server where there is no contention for the queue. However, this places a significant prerequisite knowledge or labour barrier for instructors, who must spend time coordinating deployment and management of compute resources. Furthermore, with the increase of virtual and hybrid teaching, where learners are located in separate physical locations, it is difficult to track student progress as efficiently as during in-person courses.<br>Findings: Originally developed by Galaxy Europe and the Gallantries project, together with the Galaxy community we have created "Training Infrastructure-as-a-Service" (TIaaS), aimed at providing user-friendly training infrastructure to the global training community. TIaaS provides dedicated training resources for Galaxy-based courses and events. Event organisers register their course, after which trainees are transparently placed in a private queue on the compute infrastructure, which ensures jobs complete quickly, even when the main queue is experiencing high wait times. A built-in dashboard allows instructors to monitor student progress.<br>Conclusions: TIaaS provides a significant improvement for instructors and learners, as well as infrastructure administrators. The instructor dashboard makes remote events not only possible but easy. Students experience continuity of learning, as all training happens on Galaxy which they can continue to use after the event. In the past 60 months, 504 training events with over 24000 learners have used this infrastructure for Galaxy training.
**Corresponding Author:**	Helena Rasche<br>Erasmus Medical Center: Erasmus MC<br>Rotterdam, Zuid Holland NETHERLANDS
**Corresponding Author Secondary Information:**	
**Corresponding Author's Institution:**	Erasmus Medical Center: Erasmus MC
**Corresponding Author's Secondary Institution:**	
**First Author:**	Helena Rasche
**First Author Secondary Information:**	
**Order of Authors:**	Helena Rasche<br>Cameron Hyde

Powered by Editorial Manager® and ProduXion Manager® from Aries Systems Corporation

	John Davis
	Simon Gladman
	Nate Coraor
	Anthony Bretaudeau
	Gianmauro Cuccuru
	Wendi Bacon
	Beatriz Serrano-Solano
	Jennifer Hillman-Jackson
	Saskia Hiltemann
	Miaomiao Zhou
	Björn Grüning
	Andrew Stubbs
**Order of Authors Secondary Information:**	
**Response to Reviewers:**	I have added the RRID and GigaDB DOIs as well as fixing the citations, hopefully.<br><br>It seems that when I attached the citations as a separate file (paper-refs.bib) they were not included in the build process? (i.e. in the previous submissions) I'm not sure why this was but it builds correctly locally, on overleaf, and on a third system. as a result I've directly included the citations in my main TeX document to work around whatever EditorialManager is doing :)<br><br>I've kept the paper-refs.bib included in case it is useful for y'all.<br><br>I have additionally included a \`main.pdf\` as the manuscript is mandatory, but it refuses to be attached properly during the revision preparation with the following error, I do not know why this is, the PDF is fine. It loads in multiple PDF viewers I have.<br><br><br>Your PDF file "main.pdf" cannot be opened and processed. Please see the<br>common list of problems, and suggested resolutions below.<br>Reason:<br>Other Common Problems When Creating a PDF from a PDF file<br>--<br>You will need to convert your PDF file to another format or fix the<br>current PDF file, then re-submit it
**Additional Information:**	
**Question**	**Response**
Are you submitting this manuscript to a special series or article collection?	No
**Experimental design and statistics**<br><br>Full details of the experimental design and statistical methods used should be given in the Methods section, as detailed in our Minimum Standards Reporting Checklist. Information essential to interpreting the data presented should be made available in the figure legends.	Yes

Have you included all the information requested in your manuscript?	
**Resources**<br><br>A description of all resources used, including antibodies, cell lines, animals and software tools, with enough information to allow them to be uniquely identified, should be included in the Methods section. Authors are strongly encouraged to cite Research Resource Identifiers (RRIDs) for antibodies, model organisms and tools, where possible.<br><br>Have you included the information requested as detailed in our Minimum Standards Reporting Checklist?	No
If not, please give reasons for any omissions below.<br><br>as follow-up to "**Resources**<br><br>A description of all resources used, including antibodies, cell lines, animals and software tools, with enough information to allow them to be uniquely identified, should be included in the Methods section. Authors are strongly encouraged to cite Research Resource Identifiers (RRIDs) for antibodies, model organisms and tools, where possible.<br><br>Have you included the information requested as detailed in our Minimum Standards Reporting Checklist?<br><br>"	I have requested an RRID and am waiting for their response. Thus it has not been included in the repository. In lieu of that I've included a zenodo DOI for that repository.
**Availability of data and materials**<br><br>All datasets and code on which the conclusions of the paper rely must be either included in your submission or	Yes

Powered by Editorial Manager® and ProduXion Manager® from Aries Systems Corporation

deposited in publicly available repositories (where available and ethically appropriate), referencing such data using a unique identifier in the references and in the "Availability of Data and Materials" section of your manuscript.<br><br>Have you have met the above requirement as detailed in our Minimum Standards Reporting Checklist?	

LaTeX - Main Document (TeX file)
Click here to access/download;LaTeX - Main Document (TeX file);main.tex

GigaScience, 2022, 1–7

doi: xx.xxxx/xxxx
Manuscript in Preparation
Technical Note

TECHNICAL NOTE

Training Infrastructure as a Service

Helena Rasche[1,2*], Cameron Hyde[3,4], John Davis[5], Simon Gladman†[6], Nate Coraor[9], Anthony Bretaudeau[7,8], Gianmauro Cuccuru[12], Wendi Bacon[9], Beatriz Serrano-Solano[10,12], Jennifer Hillman-Jackson[11], Saskia Hiltemann[1], Miaomiao Zhou[2], Björn Grüning[12‡] and Andrew Stubbs[1‡]

[1]Department of Pathology and Clinical Bioinformatics, Erasmus Medical Center, Wytemaweg 80, 3015 CN, Rotterdam, The Netherlands and [2]School of Life Sciences and Technology, Avans University of Applied Sciences, Lovensdijkstraat 63, 4818 AJ Breda, the Netherlands and [3]Queensland Cyber Infrastructure Foundation Ltd., The University of Queensland, St. Lucia, QLD 4072 Australia and [4]University of the Sunshine Coast, 4 Locked Bag, Maroochydore, QLD 4558 Australia and [5]Department of Biology, Johns Hopkins University, Baltimore, MD, United States and [6]Melbourne Bioinformatics, The University of Melbourne, Australia. and [7]IGEPP, INRAE, Institut Agro, Univ Rennes, 35000, Rennes, France and [8]GenOuest Core Facility, Univ Rennes, Inria, CNRS, IRISA, 35000, Rennes, France and [9]School of Life, Health & Chemical Sciences; The Open University, Milton Keynes, UK and [10]Euro-Bioimaging ERIC Bio-Hub, EMBL, Meyerhofstrasse 1, 69117 Heidelberg, Germany and [11]Department of Biochemistry and Molecular Biology, Eberly College of Science, The Pennsylvania State University, PA, United States and [12]Bioinformatics Group, Department of Computer Science, University of Freiburg, 79110 Freiburg im Breisgau, Germany

*e.rasche@erasmusmc.nl
†Deceased.
‡These authors contributed equally.

Abstract

Background: Hands-on training, whether in bioinformatics or other domains, often requires significant technical resources and knowledge to set up and run. Instructors must have access to powerful compute infrastructure that can support resource-intensive jobs running efficiently. Often this is achieved using a private server where there is no contention for the queue. However, this places a significant prerequisite knowledge or labour barrier for instructors, who must spend time coordinating deployment and management of compute resources. Furthermore, with the increase of virtual and hybrid teaching, where learners are located in separate physical locations, it is difficult to track student progress as efficiently as during in-person courses.
Findings: Originally developed by Galaxy Europe and the Gallantries project, together with the Galaxy community we have created "Training Infrastructure-as-a-Service" (TIaaS), aimed at providing user-friendly training infrastructure to the global training community. TIaaS provides dedicated training resources for Galaxy-based courses and events. Event organisers register their course, after which trainees are transparently placed in a private queue on the compute infrastructure, which ensures jobs complete quickly, even when the main queue is experiencing high wait times. A built-in dashboard allows instructors to monitor student progress.
Conclusions: TIaaS provides a significant improvement for instructors and learners, as well as infrastructure administrators. The instructor dashboard makes remote events not only possible but easy. Students experience continuity of learning, as all training happens on Galaxy which they can continue to use after the event. In the past 60 months, 504 training events with over 24000 learners have used this infrastructure for Galaxy training.

Key words: Galaxy; Training; Teaching; Remote Training

Key Points

- The private queue offered by most TIaaS deployments ensures that courses run smoothly and efficiently.
- Infrastructure is generally complicated and difficult to setup, and at cross purposes to instructors' main focus.
- TIaaS provides "one click" infrastructure for instructors that simplifies hosting courses.
- The dashboard enables remote training, allowing instructors to follow student progress.

Findings

Training Infrastructure as a Service (TIaaS) has been in development since 21 June 2018, and three days later became a production service at Galaxy Europe on 24 June. Here we present the development and rationale for implementing this service.

Background

With the large volume of bioinformatics data being generated, the availability of training for bioinformaticians and data scientists is not keeping up, resulting in a training gap [1].

The Galaxy platform [2] provides infrastructure suitable not only for data analysis, but also for conducting trainings, as it provides a user-friendly web-based interface to command-line analysis tools. Teaching with Galaxy significantly decreases infrastructure preparation time for instructors [3]. With a wide range of tools (8,000+) across a broad range of scientific domains, and pre-existing popularity within the life sciences community, Galaxy is an ideal platform for training [4, 3].

In an attempt to address the training gap, the Galaxy community has, over the past several years, developed a large number of hands-on tutorials (300+)–covering bioinformatics and beyond–and made these materials FAIR [5, 6], and publicly available on the Galaxy Training Network (GTN) repository [7]. In order to run these tutorials at scale, one often needs access to significant resources. For example, the GTN's most popular tutorial, "Reference-based RNA-Seq data analysis", uses the STAR aligner[8]. While such an ultra-fast aligner is ideal during training, as it reflects real-world analysis, it also consumes ≈32 GB of RAM at minimum[1]. Individual STAR jobs might execute successfully and quickly, however the infrastructure remains a limiting factor for events with a large number of participants, especially if the class is to remain on schedule. When jobs must queue due to throughput limitations, this negatively impacts a training's timeline, to the detriment of learners.

While the instructor could potentially deploy their own private infrastructure, this requires additional knowledge, time, energy, and funds, all of which are significant barriers for bioinformatics instructors preparing to teach a course. There are numerous attempts to decrease the effort required to deploy a Galaxy server such as Laniakea[9], CloudLaunch[10], and AnVIL[11], however most of these require access to a public or private cloud and a compute budget. Given the presence of numerous large Galaxy deployments that offer compute and data storage for free, a solution that can leverage these existing centres of Galaxy and system administration experience is highly desirable.

Lastly, with the recent increase of remote and hybrid training–where an instructor is streamed live to multiple locations–due to the COVID-19 pandemic, tracking student progress in a remote learning setting has became a significant issue. During one of the initial Gallantries project's [12] hybrid training events, with three classrooms spread across Europe, we discovered that staying updated on student progress was one of the most significant pain points. Normally instructors of hands-on lessons tend to wander around the classroom to check that students are not encountering difficulties, or use the Carpentries-style [13, 14] method of red and green sticky notes to let students communicate whether things are going well or poorly. In hybrid events this progress tracking is more difficult as on-site staff need to survey the room and report back centrally to the instructor, and is near impossible in fully remote training events such as have been more prevalent during the last 3 years of the pandemic [15].

1 It uses 90 GB of RAM in the default configuration on UseGalaxy.eu. Many tools have similar requirements; on UseGalaxy.eu, 83 tools require >64 GB RAM, 151 require >32 GB, a limiting factor especially for smaller training infrastructures. Even with a large computer cluster, even moderate class sizes of 20-40 can still consume all of the available overhead.

Results

We initially developed Training Infrastructure as a Service for the European Galaxy server [16], to solve the challenge of ensuring we could quickly setup a private queue for a single course or workshop. We achieved this by segregating student jobs onto a separate and dedicated group of compute nodes, based on their membership in a specific group in Galaxy.

We subsequently made TIaaS available for any training organizers to request free of charge. By re-using an existing public Galaxy server such as Galaxy Europe, which is backed by significant compute resources, the barriers for course organizers around infrastructure setup and maintenance costs of hosting a training event are removed. This centralisation also reduced the infrastructure requirements, as training events are not highly concurrent and can share the same hardware when not running simultaneously.

When using TIaaS for a training event, a live dashboard (Figure 1) becomes available to instructors, showing the status of participants' jobs, providing visibility into student progress and enabling instructors to flag potential issues that may benefit from additional discussion with learners. We have shared this service with the Galaxy training community to overwhelmingly positive feedback, anecdotally [17].

Deployment

The TIaaS system can be deployed on any Galaxy server, and by its design is extremely flexible, allowing Galaxy administrators to customize the settings to fit their needs and compute infrastructure. TIaaS is currently deployed on all 3 major public Galaxy servers (Galaxy EU, Galaxy Australia, and Galaxy US), and numerous other smaller servers in public and private deployments. As compute infrastructures can be highly heterogeneous we do not prescribe a single preferred method in which to preference training jobs. As a result administrators have generally allocated private resources so jobs can run without delay, with the exception of one site which preferences jobs by scheduling rules.

TIaaS provides a good separation of responsibilities between

Compiled on: June 7, 2023.
Draft manuscript prepared by the author.

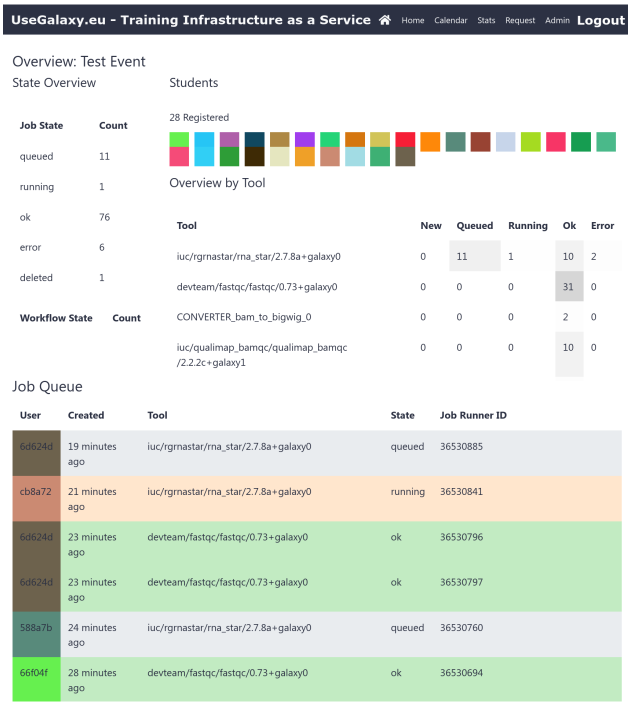

Figure 1. The top portion of the training dashboard page shows the status of the jobs in the past hours. A greyscale heatmap of the tools which were run indicates if everything is running smoothly or if there is anything the instructors should look into. As learners follow along and run different tools, these show up immediately in the dashboard, allowing instructors to identify if everyone has started or finished a specific step. The bottom portion shows the rest of the training dashboard, which lists jobs and workflows that were run, chronologically, colour-coded first by user, and second by the job status. Randomised colours and identifiers are used to protect user privacy.

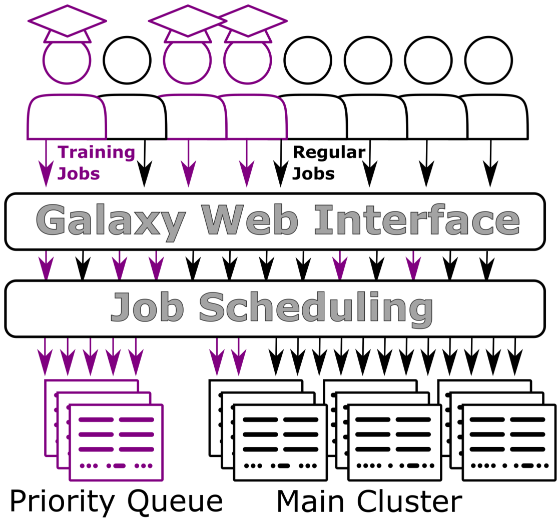

Figure 2. Schematic of the idealised TIaaS queuing system. Jobs are processed by the same Galaxy server, but when those jobs come from users in the training group, they receive special handling. These jobs are allowed to run on the private training resources (purple). If the training resource is full, these jobs can spill over to the main queue if necessary.

instructors who are teaching and the server administrators responsible for Galaxy and the compute infrastructure, rather than requiring either group to be cross-trained.

Development

To create TIaaS (RRID: SCR_023200), we implemented two components: a web service, and a default set of Galaxy job scheduling rules, which function together to present a private queue for users in specific Galaxy user groups. The web service enables registering requests for resources and an approval workflow for administrators. Additionally it handles creating groups in Galaxy and adding members to those groups as needed.

The registration form provided by the web service allows instructors to submit requests for TIaaS resources. Anyone wishing to host a training or workshop occurring on the Galaxy platform is welcome to do so as there is no formal qualification process for Galaxy instructors. Within the TIaaS request form they are asked to provide information about the training materials they will use, and the expected number of participants. TIaaS coordinators or system administrators review these requests, using information about the class size, the tools used in the training materials, as well as the resource allocations of those tools on the infrastructure, to estimate the required compute resources.

A typical request timeline looks like an instructor submitting a request with one or more weeks advance notice, as the TIaaS service will automatically reject requests that are made within a configurable length of time before the start of the course. This feature was added as a result of too many last-minute requests placing undue burden on administrators. In exceptional circumstances, administrators can manually add a training at a specific date. The vast majority of approved TIaaS requests are accepted (n=371/397), with most requests happening 7-14 days ahead of the event (n=75), while many occur in the last week (n=62), or even three (n=65), four (n=46), or five (n=39) weeks in advance.

If resources are available and any other site-specific criteria are met (e.g. any legal restrictions on what sort of trainings can be provided on grant funded infrastructure), then the training can be approved. Next, administrators (optionally) deploy additional private compute resources, or re-allocate existing resources to course usage. Administrators can then provide instructors with a URL such as `/join-training/test`[18], which the instructor can share with learners.

Training participants access this URL at the start of the event, after which they are automatically registered in the TIaaS system without further user interaction and without instructors needing to manually manage group membership. This aids in user privacy as the instructor does not need to collect user emails to manage their group, and learners can opt-in to joining the training group.

The job scheduler, once aware of the training group, will place any job run by someone in that training onto the private training nodes (Figure 2).

During the course, instructors have access to the course dashboard, visualising the progress of participants (Figure 1), significantly improving the ability of instructors to monitor progress of the learners, especially in situations involving remote participants. The dashboard provides instantaneous, aggregated, and pseudonymised feedback for the instructors into how the learners are progressing. It has also simplified progress tracking in hybrid trainings, which were previously very labour intensive due to the necessity of maintaining insight into potential issues across multiple locations. This required per-site helpers to regularly update the instructor as to how participants were progressing. With the training dashboard however, the instructor is no longer dependent on these communications from the satellite locations, but can monitor progress via the dashboard themselves, in real-time. Instructors can see which analysis steps are completed, and by how many of the participants. Whenever there are any issues (e.g. failed jobs), they can use this information to decide whether they need to pause or re-explain the step in more detail.

The most similar system the authors could find, that could be used for the same goal of monitoring student progress, is currently implemented in Nextflow. "Nextflow Tower"[19], which permits launching and subsequently monitoring pipelines, and could be

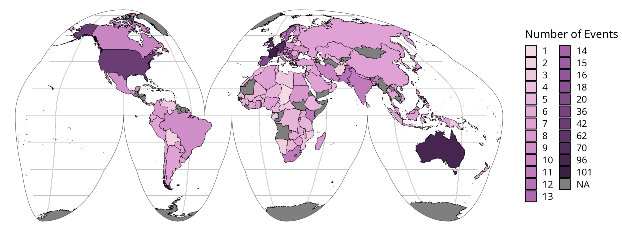

Figure 3. Map of countries targeted by TIaaS events. This combines two datasets: the statistics provided by the APIs of the four discussed TIaaS servers, and a set of corrections from course registration data for the Smörgåsbord event series. This correction is needed as the authors did not sufficiently fill out the TIaaS form when they requested resources for the Smörgåsbord event, choosing to specify only a single country, which would otherwise result in potential under-counting of countries actually targeted by TIaaS managed events.

used to cover a similar case of making sure students meet certain progress markers. However, given that it works at the workflow level and not the individual step level, it may be less suited to the sort of *ad hoc* analysis skills that are commonly taught using Galaxy, and more suited to either advanced students or those trainings which involve running pre-defined workflows. Snakemake has a similar, albeit single-user project called Panoptes that provides similar workflow tracking[20], with the same downsides as Nextflow Tower, relative to TIaaS.

Usage

Since the introduction of TIaaS in 2018, it has seen nearly constant use with 504 trainings occurring on the platform, all across the world (Figures 3 & 4). Everything from one-day workshops for bioinformaticians to multi-month courses for high school and university students have all been hosted by these four TIaaS deployments, covering topics as wide-ranging as SARS-CoV-2 analysis, Imaging, Proteomics, and Machine Learning. All of this infrastructure has been provided for free across these four instances in the EU, France, the Americas, and Australia, thanks to the various grants supporting their associated Galaxy deployments.

Class sizes have ranged considerably from the median of 25 participants (IQR=19) to a maximum of 1500 registrants for a fully asynchronous (self-paced) course. Most courses were short training events with a median of two days, however some ran for multiple months like a number of high school or university courses which used TIaaS over the entire semester. The variability in administrator deployments of TIaaS can allow it to accommodate a wide range of teaching scenarios; for some courses large resources may be allocated like the Galaxy Community Conferences where the big three Galaxies configured TIaaS with considerable resources to permit local and remote synchronous training, all the way to semester-long courses which may not necessitate a large allocation.

TIaaS has been successfully scaled to extremely large and highly geographically distributed events. The GTÑ project successfully used it for a Spanish language bioinformatics course spanning the Americas and Europe[21], while the two Smörgåsbord events used TIaaS for a week long, global, asynchronous course with trainees across 111 countries[22].

In a hackathon environment, TIaaS has allowed large dataset (single cell RNA-seq) manipulation within group projects in remote courses with up to 30 participants performing unique analyses[23]. It has successfully supported an introduction to bioinformatics course at a remote-learning, entrance-exam-free alternative education institution (The Open University) as well as industry courses, allowing them to test out Galaxy as a collaborative working environment before making decisions on consortium platform use.

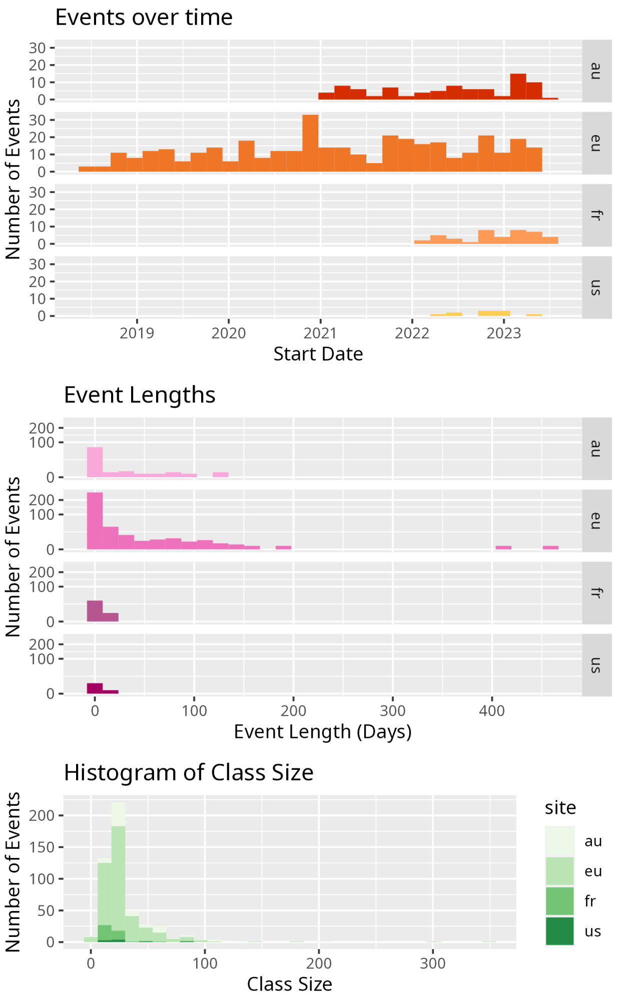

Figure 4. Since its introduction it has grown into a well-used service over the past four years. There have been 438 training events, primary hosted by the Australian and European instances which are both very involved in training. Event length distribution in days is extremely heavily skewed to very short events, with a long tail of semester-long courses using the platform. Event sizes show a similar distribution, most classes are small, while seven extremely large courses (>=500 participants) were filtered from this graph as outliers. These courses are more like MOOCs than traditional in person courses.

Methods

Implementation

TIaaS was written in Python with the Django framework [24]. It has been designed from the start to have a very limited scope: provide a form to register events, an approval flow for administrators, management of user groups and roles in the Galaxy database, and the status dashboard. Service metrics are exposed via a Prometheus [25] endpoint at `/tiaas/metrics`[26], for visualisation and alerting.

Instructors can visit (`/tiaas/new`) to register a new event and request resources. When submitted, this form is stored in the associated database and administrators can view the requested training events and approve or reject them using the built in Django admin interface. When users visit their training URL (`/join-training/<id>`) the system accesses their Galaxy session cookie, which is present as TIaaS is deployed at a path below Galaxy, and decodes it, turning it into a Galaxy identity. This identity is then automatically associated with a Galaxy group named after the training (e.g. `training-<id>`) which is created on demand.

When visiting the dashboard (Figure 1), the training ID is extracted from the URL (e.g. “test” from https://usegalaxy.eu/

join-training/test/status), and all jobs, in the past 0-6 hours, from those users are presented in a pseudonymised manner.

Overview Pages

Information over the status of the TIaaS system is provided via the interface. The calendar page made with Vue.js [27] (e.g. https://usegalaxy.eu/tiaas/calendar/) and shows upcoming training events, as well as their details if one is logged in as a TIaaS administrator. This is complemented by the stats view (e.g. https://usegalaxy.eu/tiaas/stats/) which shows overall system statistics giving funding agencies and staff a live view of the impact their service is having on the global community.

Scheduling

When a job is submitted by a user in a training group, the Galaxy instance's job scheduling system reads the user's groups and roles, and if any of these include something prefixed with `training-`, then this is converted to a job scheduler specific requirement string (Figure 5, 6). Ideally these are scheduled to prefer training nodes, and spill over to the main queue if training nodes are full, but this feature is dependent on specific scheduler capabilities.

In HTCondor this can be accomplished by preventing regular jobs from running on training nodes (e.g. a Node's configuration including `Requirements=(GalaxyGroup == training-nld) || (GalaxyGroup == training-aus)`), and then allowing training jobs to run on training nodes, and preferring those nodes via configuration (e.g. a submit description including `+Group="training-aus, training-nld"`)

Slurm, in contrast, requires either using TPV's notion of machine tags to separate jobs into those specific groups of machines, or simply manually selecting a reservation in which to run the training jobs, with `-partition=training-nld`.

```
def queue_job(job, user):
    job.cluster = 'main'

    if inTrainingGroup(user):
        training = getTrainingGroup(user)
        job.cluster = training

    return job
```

Figure 5. Pseudocode representing how TIaaS jobs are typically processed and allocated to a private queue.

Or, rewritten for the modern Total Perspective Vortex (TPV)[28] scheduler that is now being used at all three large UseGalaxy servers:

```
roles:
  training.*:
    scheduling:
      require:
        - training
```

Figure 6. YAML formatted TPV configuration that schedules jobs coming from users with a training role to any machines labelled as training nodes.

Flexible Deployments

As the Galaxy community has largely settled on Ansible for deployment of Galaxy, and related components, an Ansible role was produced for deploying the TIaaS Service. A few known deployments make their configuration public, and as such we can see what choices each administrator made. One of the motivating factors in TIaaS' design was such flexibility, this advantage is seen directly in those deployments.

Galaxy Europe uses it with HTCondor, and job rules that allow spill over to the main cluster; new machines are brought up in an OpenStack cluster specifically for training events and destroyed afterwards. Each Machine is tagged with an HTCondor attribute indicating which training it belongs to, and the job rules[2] use that to enable access to those machines, and a preference for them.

Galaxy Australia has a separate "training cluster" in their OpenStack deployment, and route all training jobs to the single shared cluster[3].

Galaxy US takes a different approach, lacking additional clusters but having an efficient queuing system that can properly pack jobs based on walltimes; they instead artificially limit the runtime, memory, and CPU resources allocated to users running jobs within a TIaaS group.

Avans Hogeschool uses TIaaS in an internal Galaxy where they provide no preferential treatment, and just use the dashboard to follow students' progress[4].

Availability of source code and requirements

- Project name: Training Infrastructure as a Service
- RRID: SCR_023200
- bio.tools ID: `tiaas`
- Project home: https://github.com/galaxyproject/tiaas2/
- Admin Training Manual: https://gxy.io/GTN:T00022
- Teacher Training Manual: https://gxy.io/GTN:T00286
- Programming Language(s): Python, Vue.js
- Operating system(s): Unix
- License: GNU AGPL-3.0

Data Availability

All code is open source and available on GitHub [29, 30]. Snapshots of our code and other data further supporting this work are openly available in the GigaScience repository, GigaDB [31].

Declarations

List of abbreviations

- TIaaS: Training Infrastructure as a Service

Ethics approval and consent to participate

Not applicable

Consent for publication

Not applicable

2 Visible in https://github.com/usegalaxy-eu/infrastructure-playbook/pull/447/files
3 Visible in https://github.com/usegalaxy-au/infrastructure/tree/57cd80030d72929c263955e895079d6ac25aa24f/files/galaxy/dynamic_job_rules/production/total_perspective_vortex, note the `training` role and destinations tagged training.
4 Visible at https://github.com/Avans-ATGM/infrastructure/commit/11fa1ed38a7ed6640eafeca2ace8bb73e189301e

Competing Interests

The authors declare that they have no competing interests.

Funding

The work is in part funded by Collaborative Research Centre 992 Medical Epigenetics (DFG grant SFB 992/1 2012), German Federal Ministry of Education and Research (BMBF grants 031 A538A/A538C RBC and 031L0101B/031L0101C de.NBI-epi, doi:10.13039/501100002347), and the National Institutes of Health, U.S. (National Human Genome Research Institute; grant 2U24HG006620, doi:10.13039/100000051).

Development work and article publication charges, are additionally funded with the support of the Erasmus+ programme of the European Union (Grant 2020-1-NL01-KA203-064717, doi:10.13039/100001501).

Author's Contributions

Author contributions, described using the CASRAI CRedIT typology (http://casrai.org/credit), are as follows:

- Conceptualization: HR, BG;
- Methodology: HR;
- Software: HR, CH, JD, AB;
- Validation: WB, BSS, JHJ, SH;
- Investigation: WB, BSS, JHJ;
- Resources: BG, SG, NC, AB, AS, BSS, JHJ;
- Writing – Original Draft Preparation: HR, BG;
- Writing – Review & Editing: HR, SH, JD, MZ;
- Visualization: HR, CH;
- Supervision: AB, BG;
- Funding Acquisition: BG, AS, SH, AB, HR.

Acknowledgements

In loving memory of Simon Gladman (1970-2022) beloved mentor and system administrator, who was instrumental in getting the second TIaaS deployment running at Galaxy Australia, proving its generalisability.

The authors would like to thank the Galaxy community for their enthusiasm for this project, and their feedback on each iteration. This project was made possible with the support of the Albert Ludwig University of Freiburg.

References

1. Attwood TK, Blackford S, Brazas MD, Davies A, Schneider MV. A global perspective on evolving bioinformatics and data science training needs. Briefings in Bioinformatics 2017 Aug;20(2):398–404. https://doi.org/10.1093/bib/bbx100.
2. Afgan E, Baker D, Batut B, Van Den Beek M, Bouvier D, Čech M, et al. The Galaxy platform for accessible, reproducible and collaborative biomedical analyses: 2018 update. Nucleic acids research 2018;46(W1):W537–W544.
3. Hiltemann S, Rasche H, Gladman S, Hotz HR, Larivière D, Blankenberg D, et al. Galaxy Training: A powerful framework for teaching! PLoS Comput Biol Computational Biology 2023 jan;19(1):e1010752. https://doi.org/10.1371%2Fjournal.pcbi.1010752.
4. Batut B, Hiltemann S, Bagnacani A, Baker D, Bhardwaj V, Blank C, et al. Community-Driven Data Analysis Training for Biology. Cell Systems 2018 Jun;6(6):752–758.e1. https://doi.org/10.1016/j.cels.2018.05.012.
5. Wilkinson MD, Dumontier M, Aalbersberg IJJ, Appleton G, Axton M, Baak A, et al. The FAIR Guiding Principles for scientific data management and stewardship. Sci Data 2016 Mar;3:160018.
6. Garcia L, Batut B, Burke ML, Kuzak M, Psomopoulos F, Arcila R, et al. Ten simple rules for making training materials FAIR. PLOS Computational Biology 2020 05;16(5):1–9. https://doi.org/10.1371/journal.pcbi.1007854.
7. Galaxy Training Materials;. https://training.galaxyproject.org.
8. Dobin A, Davis CA, Schlesinger F, Drenkow J, Zaleski C, Jha S, et al. STAR: ultrafast universal RNA-seq aligner. Bioinformatics 2012 Oct;29(1):15–21. https://doi.org/10.1093/bioinformatics/bts635.
9. Tangaro MA, Donvito G, Antonacci M, Chiara M, Mandreoli P, Pesole G, et al. Laniakea: an open solution to provide Galaxy "on-demand" instances over heterogeneous cloud infrastructures. GigaScience 2020 apr;9(4). https://doi.org/10.1093%2Fgigascience%2Fgiaa033.
10. Afgan E, Lonie A, Taylor J, Goonasekera N. CloudLaunch: Discover and deploy cloud applications. Future Generation Computer Systems 2019 may;94:802–810. https://doi.org/10.1016%2Fj.future.2018.04.037.
11. Schatz MC, Philippakis AA, Afgan E, Banks E, Carey VJ, Carroll RJ, et al. Inverting the model of genomics data sharing with the NHGRI Genomic Data Science Analysis, Visualization, and Informatics Lab-space. Cell Genomics 2022 jan;2(1):100085. https://doi.org/10.1016%2Fj.xgen.2021.100085.
12. Gallantries T, Bringing galaxy and the carpentries together;. Online; accessed 21 October 2022. https://gallantries.github.io/.
13. Carpentries T, The Carpentries;. Online; accessed 21 October 2022. https://carpentries.org/.
14. Wilson G. Software Carpentry: lessons learned. F1000Research 2016 Jan;3:62. https://doi.org/10.12688/f1000research.3-62.v2.
15. Serrano-Solano B, Föll MC, Gallardo-Alba C, Erxleben A, Rasche H, Hiltemann S, et al. Fostering accessible online education using Galaxy as an e-learning platform. PLoS Computational Biology 2021 may;17(5):e1008923. https://doi.org/10.1371%2Fjournal.pcbi.1008923.
16. Galaxy Europe;. Accessed: 2023-06-07. https://usegalaxy.eu/.
17. Community G, TIaaS Feedback;. Online; accessed 21 October 2022. https://galaxyproject.eu/news?tag=TIaaS.
18. Galaxy Europe: Join Training Test;. Accessed: 2023-06-07. https://usegalaxy.eu/join-training/test.
19. Nextflow, Nextflow Tower;. Online; accessed 15 November 2022. https://tower.nf/.
20. Organization P, Panoptes;. Online; accessed 15 November 2022. https://github.com/panoptes-organization/panoptes.
21. Spanscriptomics: Análisis de células únicas usando Galaxy;. Accessed: 2023-01-25. https://gallantries.github.io/galaxy-workshop/events/spanscriptomics/.
22. GTN Smörgåsbord: A Global Galaxy Course;. Accessed: 2023-01-25. https://gallantries.github.io/posts/2021/03/01/sm%C3%B6rg%C3%A5sbord/.
23. Bacon W, Holinski A, Pujol M, Wilmott M, and SLM. Ten simple rules for leveraging virtual interaction to build higher-level learning into bioinformatics short courses. PLoS Computational Biology 2022 jul;18(7):e1010220. https://doi.org/10.1371%2Fjournal.pcbi.1010220.
24. Django (Version 3.1) [Computer Software];. https://www.djangoproject.com/.
25. Rabenstein B, Volz J. Prometheus: A Next-Generation Monitoring System (Talk). Dublin: USENIX Association; 2015. .
26. Galaxy Europe: TIaaS: Prometheus Metrics;. Accessed: 2023-06-07. https://usegalaxy.eu/tiaas/metrics.

27. Vue.js, The Progressive JavaScript Framework;. Accessed: 2023-01-25. https://vuejs.org/.
28. Total Perspective Vortex;. Accessed: 2023-01-25. https://github.com/galaxyproject/total-perspective-vortex/.
29. galaxyproject/tiaas2: Reusable Training Infrastructure as a Service;. Accessed: 2023-06-07. https://github.com/galaxyproject/tiaas2/.
30. galaxyproject/ansible-tiaas2: Ansible role to install TIaaS;. Accessed: 2023-06-07. https://github.com/galaxyproject/ansible-tiaas2/.
31. Helena R, Cameron H, John D, Simon G, Nate C, Anthony B, et al., Supporting data for "Training Infrastructure as a Service". GigaScience Database; 2023. http://gigadb.org/dataset/102406.

Manuscript Click here to access/download;Manuscript;main.pdf

Your PDF file "main.pdf" cannot be opened and processed. Please see the common list of problems, and suggested resolutions below.

Reason:

Other Common Problems When Creating a PDF from a PDF file

You will need to convert your PDF file to another format or fix the current PDF file, then re-submit it.
