# Supplementary material for: Training Infrastructure as a Service
Source: Gigascience. 2023 Jul 3;12:giad048. doi: 10.1093/gigascience/giad048 (PMC10316688; doi:10.1093/gigascience/giad048)
Supplement: giad048_Response_to_Reviewer_Comments_Original_Submission [file giad048_response_to_reviewer_comments_original_submission.pdf]

Author's Response To Reviewer Comments

Close

GigaScience Editors

We wanted to extend a special thank you to Dr. Nogoy and the editorial team for the very generous review timeline. It was instrumental in maintaining my sanity as we ran our third large training course atop tiaas with 3300 registered participants. It gave me time to priotise the course and address the comments once the course was over and I really sincerely appreciate it.

Reviewer 1

Thank you Dr. Ahmed, for your valuable comments. We are very proud to be able to share these resources with the world and hopefully it works to undo some of the effects of historical and current wealth centralisation in HICs, by providing these resources for free to LMICs. Likewise, hopefully as compute resources grow in LMICs we'll see more TIaaS deployments there. It's not a perfect solution by far, but it's something this author can push for within her small corner of bioinformatics. Thank you.

Reviewer 2

Thank you, Dr. Ryder, for your useful feedback!

We unfortunately do not have extensive data on the timeline, from submission to acceptance, of a typical TIaaS request.
The database records simply lack an "approved at" date. That said checking in the European TIaaS database for all approved trainings, we can see that the majority of them (301/397) were requested between 0-36 days in advance of a course.

```
( 0.000, 18.467) n=198 *************************************************
[ 18.467, 36.933) n=103 *************************
[ 36.933, 55.400) n=32 ********
[ 55.400, 73.867) n=15 ***
[ 73.867, 92.333) n=8 **
[ 92.333, 110.800) n=6 *
[ 110.800, 129.267) n=0
[ 129.267, 147.733) n=1
[ 147.733, 166.200) n=1
[ 166.200, 184.667) n=1
[ 184.667, 203.133) n=0
[ 203.133, 221.600) n=0
[ 221.600, 240.067) n=0
[ 240.067, 258.533) n=0
[ 258.533, 277.000) n=0
[ 277.000, 295.467) n=1
```

The database did include negative values which were filtered out (n=5), those likely consist of backdated trainings, or where the teacher has requested a training that is already in progress. By default the TIaaS service blocks those requests, as administrators need time to prepare them. Additionally there is a long tail on this distribution which is trimmed for legibility. One very prepared person requested it 277 days in advance!

We have included a textual representation of the above in the development section, as a result of this

feedback.

Additionally we have replaced the median and SD measurements with median and IQRs instead. A class size chart has been included, thank you for this suggestion.

> I'm not sure why 'Findings' appears before 'Background' - perhaps an editing error?

Findings was the section, Background and Results are subsections thereof. I have added an introductory sentence in the Findings section to separate the two headings, and introduce the background & results section, which hopefully corrects this visual oddity:

> Training Infrastructure as a Service (TIaaS) has been in development since 21 June 2018, and three days later became a production service at Galaxy Europe on 24 June. Here we present the development and rationale for implementing this service.

Lastly, thank you for the additional spelling and grammatical corrections.

Close
