# Supplementary material for: Training Infrastructure as a Service
Source: Gigascience. 2023 Jul 3;12:giad048. doi: 10.1093/gigascience/giad048 (PMC10316688; doi:10.1093/gigascience/giad048)
Supplement: giad048_Response_to_Reviewer_Comments_Revision_1 [file giad048_response_to_reviewer_comments_revision_1.pdf]

Author's Response To Reviewer Comments

Close

I have added the RRID and GigaDB DOIs as well as fixing the citations, hopefully.

It seems that when I attached the citations as a separate file (paper-refs.bib) they were not included in the build process? (i.e. in the previous submissions) I'm not sure why this was but it builds correctly locally, on overleaf, and on a third system. as a result I've directly included the citations in my main TeX document to work around whatever EditorialManager is doing :)

I've kept the paper-refs.bib included in case it is useful for y'all.

I have additionally included a `main.pdf` as the manuscript is mandatory, but it refuses to be attached properly during the revision preparation with the following error, I do not know why this is, the PDF is fine. It loads in multiple PDF viewers I have.

Your PDF file "main.pdf" cannot be opened and processed. Please see the
common list of problems, and suggested resolutions below.
Reason:
Other Common Problems When Creating a PDF from a PDF file

You will need to convert your PDF file to another format or fix the
current PDF file, then re-submit it

Close
