# Supplementary material for: Training Infrastructure as a Service
Source: Gigascience. 2023 Jul 3;12:giad048. doi: 10.1093/gigascience/giad048 (PMC10316688; doi:10.1093/gigascience/giad048)
Supplement: giad048_Reviewer_1_Report_Original_Submission — Azza Ahmed -- 3/26/2023 Reviewed [file giad048_reviewer_1_report_original_submission.pdf]

Reviewer Report

Title: Training Infrastructure as a Service

Version: Original Submission **Date:** 3/26/2023

Reviewer name: Azza Ahmed

Reviewer Comments to Author:

The paper is well-written and neatly reports on the development of Training-Infrastructure-as-a-Service (TIaaS), a free infrastructure resource originally developed by Galaxy Europe and the Gallantries project together with the Galaxy community. TIaaS is a step towards democratizing bioinformatics training, where infrastructure can be a major barrier- even in advanced and well-developed countries.I specially appreciate the value of this resource for instructors and students in low and middle income countries where infrastructure limitations may be exacerbated by the availability of well-trained system administrators able to cater specific training needs. It was indeed gratifying to see training events using TIaaS in such countries in the figure 3 map- especially that it is not clear TIaaS is deployed in such counties. The utility of the resource is self-evident: 438 training events in 48 months targeting > 19000 students. Thus, overall, I congratulate the authors for the success of their project, and the community for having such a great _free_ resource at their disposal.

Level of Interest

Please indicate how interesting you found the manuscript: Choose an item.

Quality of Written English

Please indicate the quality of language in the manuscript: Choose an item.

Declaration of Competing Interests

Please complete a declaration of competing interests, considering the following questions:

- Have you in the past five years received reimbursements, fees, funding, or salary from an organisation that may in any way gain or lose financially from the publication of this manuscript, either now or in the future?
- Do you hold any stocks or shares in an organisation that may in any way gain or lose financially from the publication of this manuscript, either now or in the future?
- Do you hold or are you currently applying for any patents relating to the content of the manuscript?
- Have you received reimbursements, fees, funding, or salary from an organization that holds or has applied for patents relating to the content of the manuscript?
- Do you have any other financial competing interests?

- Do you have any non-financial competing interests in relation to this paper?

If you can answer no to all of the above, write 'I declare that I have no competing interests' below. If your reply is yes to any, please give details below.

I declare that I have no competing interests

I agree to the open peer review policy of the journal. I understand that my name will be included on my report to the authors and, if the manuscript is accepted for publication, my named report including any attachments I upload will be posted on the website along with the authors' responses. I agree for my report to be made available under an Open Access Creative Commons CC-BY license (http://creativecommons.org/licenses/by/4.0/). I understand that any comments which I do not wish to be included in my named report can be included as confidential comments to the editors, which will not be published.

Choose an item.

To further support our reviewers, we have joined with Publons, where you can gain additional credit to further highlight your hard work (see: https://publons.com/journal/530/gigascience). On publication of this paper, your review will be automatically added to Publons, you can then choose whether or not to claim your Publons credit. I understand this statement.

Yes Choose an item.
