# Supplementary material for: Training Infrastructure as a Service
Source: Gigascience. 2023 Jul 3;12:giad048. doi: 10.1093/gigascience/giad048 (PMC10316688; doi:10.1093/gigascience/giad048)
Supplement: giad048_Reviewer_2_Report_Original_Submission — Elizabeth Ryder -- 4/3/2023 Reviewed [file giad048_reviewer_2_report_original_submission.pdf]

Reviewer Report

Title: Training Infrastructure as a Service

Version: Original Submission **Date:** 4/3/2023

Reviewer name: Elizabeth Ryder

Reviewer Comments to Author:

This technical note is an informative explanation of Training-Infrastructure-as-a-Service, which is a free service available to facilitate Galaxy training sessions. The service provides an easy way for instructors to set up infrastructure for trainings, enables learners to make progress through the training without long waiting times, and includes a dashboard through which instructors can easily monitor progress of learners. The article provides data showing the large number of events and locations that have benefited from using TIaaS. Because of the utility and general applicability of TIaaS, the article will be of interest to the readers of GigaScience.Minor suggestions:In the Development section: As a practical matter, it would be useful to know the typical timeline for approval of a training session. Also, can anyone who uses Galaxy become an instructor and request this service?In the Usage section, there is a sentence that reads, 'Class sizes have ranged considerably, from the median of 25 participants (std. dev 121) to a maximum of 1500 registrants for afully asynchronous (self-paced) course.' It's a little unusual to talk about a median and standard deviation, since medians are non-parametric measures and SDs are parametric and measured with respect to the mean. I'd suggest using the median and interquartile range instead. I think a histogram of class size distribution would be informative, similar to the event distributions in Fig. 4.Grammatical / spelling errors:I'm not sure why 'Findings' appears before 'Background' - perhaps an editing error?p. 2'a limiting factor for events with large number of participants, 'should read'with a large number of participants''by it's design'should read'by its design''which to to preference'should read'which to preference'p.4'univeristy'should read'university'p.5This sentence is hard to scan as written; I think it needs a semi-colon after 'cluster' to make sense.Galaxy Europe uses it with HTCondor, and job rules that allowspill over to the main cluster, new machines are brought up in anOpenStack cluster specifically for training events and destroyed afterwards.

Level of Interest

Please indicate how interesting you found the manuscript: Choose an item.

Quality of Written English

Please indicate the quality of language in the manuscript: Choose an item.

Declaration of Competing Interests

Please complete a declaration of competing interests, considering the following questions:

- Have you in the past five years received reimbursements, fees, funding, or salary from an organisation that may in any way gain or lose financially from the publication of this manuscript, either now or in the future?
- Do you hold any stocks or shares in an organisation that may in any way gain or lose financially from the publication of this manuscript, either now or in the future?
- Do you hold or are you currently applying for any patents relating to the content of the manuscript?
- Have you received reimbursements, fees, funding, or salary from an organization that holds or has applied for patents relating to the content of the manuscript?
- Do you have any other financial competing interests?
- Do you have any non-financial competing interests in relation to this paper?

If you can answer no to all of the above, write 'I declare that I have no competing interests' below. If your reply is yes to any, please give details below.

I declare that I have no competing interests

I agree to the open peer review policy of the journal. I understand that my name will be included on my report to the authors and, if the manuscript is accepted for publication, my named report including any attachments I upload will be posted on the website along with the authors' responses. I agree for my report to be made available under an Open Access Creative Commons CC-BY license (http://creativecommons.org/licenses/by/4.0/). I understand that any comments which I do not wish to be included in my named report can be included as confidential comments to the editors, which will not be published.

Choose an item.

To further support our reviewers, we have joined with Publons, where you can gain additional credit to further highlight your hard work (see: https://publons.com/journal/530/gigascience). On publication of this paper, your review will be automatically added to Publons, you can then choose whether or not to claim your Publons credit. I understand this statement.

Yes Choose an item.
